# Mechanisms and consequences of mRNA destabilization during viral infections

**DOI:** 10.1186/s12985-024-02305-1

**Published:** 2024-02-06

**Authors:** Soraya I. Shehata, J. Monty Watkins, James M. Burke, Roy Parker

**Affiliations:** 1 Department of Molecular, Cellular, and Developmental Biology, University of Colorado Boulder, Boulder, CO USA; 2 Medical Scientist Training Program, University of Colorado Anschutz Medical Campus, Aurora, CO USA; 3 Department of Molecular Medicine, The Herbert Wertheim UF Scripps Institute for Biomedical Innovation and Technology, Jupiter, FL USA; 4 Department of Immunology and Microbiology, The Herbert Wertheim UF Scripps Institute for Biomedical Innovation and Technology, Jupiter, FL USA; 5 Skaggs Graduate School of Chemical and Biological Sciences, The Scripps Research Institute, Jupiter, FL USA; 6 Department of Biochemistry, University of Colorado Boulder, Boulder, CO USA; 7 Howard Hughes Medical Institute, University of Colorado Boulder, Boulder, CO USA

**Keywords:** Host shutoff, SARS-CoV-2, Translation, RNA decay, RNA binding proteins

## Abstract

During viral infection there is dynamic interplay between the virus and the host to regulate gene expression. In many cases, the host induces the expression of antiviral genes to combat infection, while the virus uses “host shut-off” systems to better compete for cellular resources and to limit the induction of the host antiviral response. Viral mechanisms for host shut-off involve targeting translation, altering host RNA processing, and/or inducing the degradation of host mRNAs. In this review, we discuss the diverse mechanisms viruses use to degrade host mRNAs. In addition, the widespread degradation of host mRNAs can have common consequences including the accumulation of RNA binding proteins in the nucleus, which leads to altered RNA processing, mRNA export, and changes to transcription.

## Introduction to host shutoff mechanisms

Numerous viruses have evolved mechanisms to disrupt host gene expression. These so called "host-shut off" systems serve two purposes. First, they allow viruses to use host cellular machinery to preferentially translate viral proteins, replicate, and exit the cell. Second, host-shut off systems can limit the host antiviral response by preventing the transcription, biogenesis, and/or translation of IFN and interferon-stimulated genes (ISGs) that can interfere with the viral replication cycle [1–4].

Different classes of virus have evolved distinct mechanisms for host shutoff. Two mechansims commonly employed by viruses are the inhibition of host translation and the degradation of host mRNAs [2, 3, 5–9]. For translation shut-off, viruses can directly target host translation initiation by interfering with initiation factors or the ribosome. For example, poliovirus cleaves eIF4G to selectively disrupt eIF4F cap-dependent translation [10, 11]. Viruses can also benefit from the host antiviral response that suppresses the bulk of host translation as well. For example, viral infection by cricket paralysis virus (CrPV) causes activation of PKR, which in turn phosphorylates eIF2α causing downregulation of cellular translation [12]. However, CrPV escapes eIF2α phosphorylation by initiating translation with an eIF2α independent IRES element [13]. The wide spectrum of viral mechanisms for repressing host translation have been reviewed [14, 15]. Viruses can also interfere with host RNA processing and thereby prevent host gene expression, exemplified by the actions of HSV-1 ICP27 [16] and NS1 [17].

Host shutoff can also be mediated by host-encoded proteins. For example, the latent endoribonuclease, ribonuclease L (RNase L), can be activated in response to viral infection when dsRNA is recognized by 2ʹ-5ʹ-oligoadenylate sythetases (OASs), which produce 2ʹ-5ʹoligo(A) [18, 19]. This 2ʹ-5ʹ-oligo(A) induces RNase L dimerization, which activates its RNase domain. Once activated, RNase L then cleaves viral RNA, as well as host cellular mRNAs, tRNAs, and rRNAs [20]. RNase L induces translational reprograming and translational shutoff as a host antiviral mechanism. [21, 22].

A number of viruses, including Herpesviridae, orthomyxyoviridae, and coronaviridae, interfere with host gene expression by triggering decay of host mRNAs [2, 3, 5, 6, 23–26]. Viral mediated mRNA decay can be linked to translation repression, proceed through activation of a host ribonuclease, or be mediated through RNase activity of a viral protein. Herein we review how viruses trigger host mRNA decay and how that process alters both viral and host gene expression.

## Herpesviruses

Herpesviruses are a family of large, enveloped, double-stranded DNA viruses that are nearly ubiquitous in the human population due to their ability to establish latent infections and therefore establish a cache of virus for future reactivation [27–29]. Latent infections are difficult for the immune system to detect, as only a small number of viral proteins are expressed [30, 31]. After reactivation of a latent stage, the infection shifts to a lytic stage, during which viruses rapidly replicate and shed.

There are nine human herpesviruses, divided into three subfamilies [31]. Alphaherpesviruses include herpes simplex virus (HSV) 1, HSV 2, and varicella zoster virus (VZV); Betaherpesviruses include human cytomegalovirus (HCMV), human herpes virus (HHV) 6A, HHV-6B, and HHV 7; and Gammaherpesvirus including Epstein Barr virus (EBV) and Kaposi’s sarcoma-associated herpesvirus (KSHV) [31]. There are several examples of host mRNA degradation within both the α-herpesvirus and γ-herpesvirus sub-families, although these viruses evolved distinct mechanisms of host shutoff that are functionally unrelated. Unlike the α- and γ-families, β-herpesviruses do not cause degradation of host mRNAs, though human cytomegalovirus (HCMV) does degrade specific host miRNAs [32].

### Alpha-herpesviruses

**Fig. 1:**
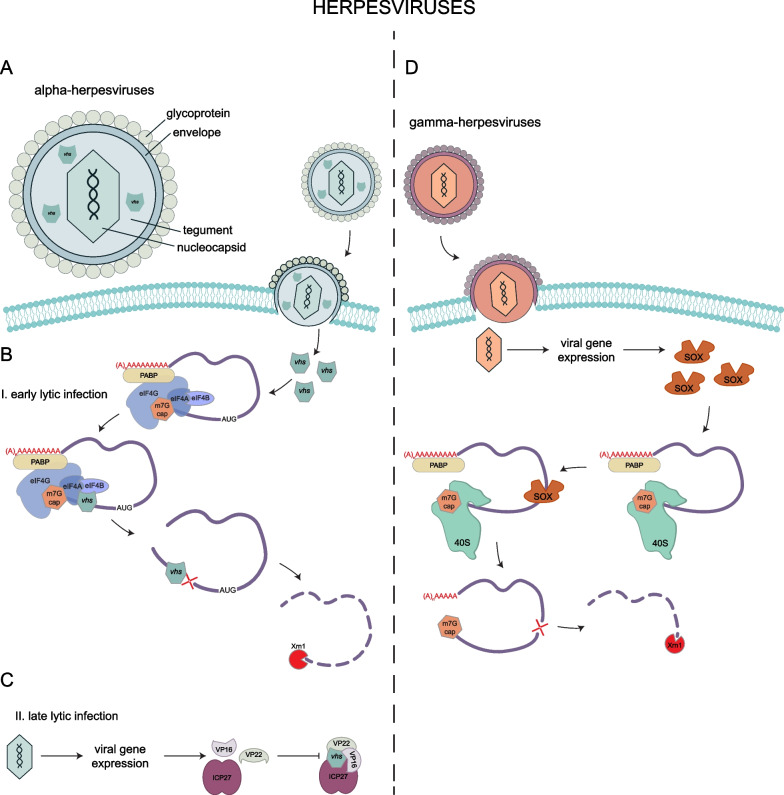
Herpesviruses host shutoff. **A** alpha-herpesviruses package the ribonuclease Vhs in their tegument, and it is released into the cytoplasm after viral entry. **B** Vhs cleaves the host mRNA. The exonuclease Xrn1 further degrades the cleavage fragments. **C** During late lytic infection, the viral proteins VP16, VP22, and ICP27 bind to and inhibit Vhs from degrading mRNAs. **D** Gamma-herpesviruses encode the SOX endonuclease, cleaves host mRNAs at an early stage of mRNA translation

There is abundant evidence that Herpes simplex virus (HSV), an α-herpesvirus, destabilizes both viral and host mRNAs through its virion host shutoff (vhs) protein [33]. Vhs is encapsulated in the HSV virion as part of the tegument [34], which is the material occupying the space between the nucleocapsid and envelope of the packaged virus (Fig. 1A). Vhs is then imported into the cell at the time of infection and within 3 h post infection, host mRNAs are largely degraded [33, 34].

Several observations show RNA degradation is dependent on Vhs expression. First, mRNA levels are decreased when cells are infected with WT virus, or transfected with WT Vhs protein, while mRNAs are more stable when cells are infected with Vhs mutant virus or transfected with mutant Vhs protein [33, 35]. Second, Vhs in virion extract degrades mRNAs in vitro without requiring any host proteins/RNases, and this degradation is inhibited by pre-incubation with an anti-Vhs antibody [34]. Third, recombinant Vhs is a endoribonuclease and degrades mRNAs in rabbit reticulocyte lysate (RRL) reactions [34]. RNA degradation is specific for mRNAs, which are cleaved near the translation initiation site, while rRNAs are unaffected by Vhs (Fig. 1B) [34].

Mechanistically, it is thought Vhs targets translating mRNAs through interactions with the eIF4F complex, and these interactions guide the specificity of Vhs targeting. Vhs associates with mRNAs through its interactions with eIF4F, eIF4H, and eIF4A [36, 37]. Point mutations and deletions in Vhs that disrupt interactions with the cap-binding complex cannot degrade mRNAs. While eIF4H is necessary for mRNA targeting, it is not sufficient, suggesting that Vhs must interact with multiple subunits of the cap-binding complex [38]. Vhs does not require mRNAs to associate with the 40S ribosome and the addition of a strong hairpin in the 5ʹ UTR that blocks 40S association is not sufficient to restore mRNA levels [39]. Vhs is also present in the nucleus, where it is proposed to recognize and cleave mRNAs with AU-rich elements (AREs) in their 3ʹ UTR through an interaction with an ARE-binding protein [40].

Counterintuitively, Vhs also degrades nearly all viral mRNAs in a manner that allows the proper timing of viral gene expression [33]. During lytic HSV infection, there are three stages of gene expression, and both transcription and mRNA stability are tightly controlled [41]. Vhs regulates the half-lives of mRNAs from each different gene class, and in cells with a vhs-mutant, the mRNAs were more stable leading to an upregulation of their protein products [35]. Vhs can also lead to translational shutofff by nuclear retention of mRNA, rather than solely through RNA degradation [42]. As lytic infection continues, Vhs activity is suppressed by viral proteins VP16, ICP27, and VP22 (Fig. 1C) [43–45]. VP22 is important for this timing, as deletion of VP22 results in delayed translational shutoff [46]. VP22 is also required for robust Vhs expression, as VP22-deletion mutants have barely detectable levels of Vhs protein, though mRNAs are still degraded [47]. This suggests that Vhs also functions to modulate the temporal pattern of viral gene expression.

### Gammaherpesviruses

Several γ-herpesviruses also destabilize host mRNAs including Kaposi’s sarcoma associated herpesvirus (KSHV), Epstein-Barr virus (EBV), and murine herpesvirus 68 (MHV68). KSHV and EBV are both oncogenic in late-stage human immunodeficiency virus infections [48, 49]. MHV68 is closely related to KSHV and EBV and is often used as a model virus since it is easier to induce lytic infection. KSHV, EBV, and MHV68 all degrade mRNAs during lytic, but not latent, infection [8, 50].

Distinct from α-herpesvirus, RNA degradation triggered by γ-herpesvirus infection is driven by a conserved alkaline exonuclease protein, named SOX in KSHV, BGLF5 in EBV, and muSOX in MHV68 (Fig. 1D) [5].These proteins are part of the PD-(D/E)XK nuclease superfamily [51, 52]. Proteins in the same alkaline exonuclease family are found across herpesviruses, including HSV gene UL12 and the HCMV gene UL98, but in those viruses the alkaline exonuclease is a DNase that resolves the branched structures formed during viral replication with no evidence for an effect on RNA [8, 53, 54]. In addition to its DNase activity, SOX has evolved a RNase activity[8]. The DNase and RNase activity in KSHV are separable by point mutations [7], but SOX binds DNA and RNA with some overlapping residues [55].

There are two pieces of evidence that show that γ-herpesviruses degrade mRNAs. First, expression of SOX, BGLF5, or muSOX in cells is sufficient to cause degradation of RNAP II-transcribed reporter mRNAs [39]. SOX separation of function mutants can isolate the DNase and host shutoff roles and therefore show that RNase activity is specific for these γ-herpesviruses [7]. Second, both SOX and BGLF5 can degrade unstructured RNA substrates in vitro [51, 56], though BGLF5 is dependent on Mn^2+^ for its Rnase activity. SOX and BGLF5 both have endonuclease activity in vitro [51, 57], and blocking exonuclease activity with 5ʹ or 3ʹ end modifications has no effect on mRNA decay. Xrn1 is required for processing the endonucleolytic cleavage fragments [55]. When Xrn1 is knocked down prior to SOX expression, reporter mRNA cleavage fragments are observed by Northern blot suggesting SOX generates limited cleavages per mRNA and Xrn1 is required to degrade the resulting RNA fragments [55].

The specificity of SOX, BGLF5, and muSOX is determined by mRNA localization and its translation status. First, SOX, BGLF5, and muSOX are all predominantly nuclear proteins with a smaller fraction in the cytoplasm [58]. The cytoplasmic fraction is thought to drive host mRNA decay because the addition of a nuclear retention signal to muSOX prevents the host shutoff function [58]. This suggests that the mRNA degradation function of mSOX occurs primarily in the cytoplasm. In addition, there is evidence that mRNAs are targeted at an early stage of translation. Reporter mRNAs with a mutant EMCV IRES that reduces ribosome subunit joining are still cleaved by SOX [55], suggesting an 80S ribosome complex is not required for SOX cleavage. Consistent with this interpretation, sucrose gradient fractionation shows that degradation intermediates accumulate with the 40S ribosome [55]. Taken together, these data suggest that the γ-herpesvirus endonuclease target cytoplasmic mRNAs at early stages of translation initiation, but the details of which stage of initiation is targeted is still unknown.

The specificity of the endonuclease cleavage sites by the SOX, BGLF5, and muSOX enzymes is not well understood. There is evidence that SOX preferentially targets specific sequences of mRNAs for degradation since deletion of five bases (TGAAG) from a GFP reporter RNA abolishes cleavage [55]. This motif is not sufficient to cause mRNA cleavage though, suggesting that perhaps there is a larger structural element that determines the SOX cleavage site, which could include these sequence motifs. There is also evidence that SOX requires stem loops and bulges to endonucleolytically cleave RNA both in vitro and in cells [59, 60], which suggests a preference for targeting unpaired nucleotides. This is consistent with the observation that mRNAs are cleaved during early translation initiation, since secondary structure is unwound to facilitate loading onto the 40S [55]. SOX was unable to process a double-stranded 51-mer of RNA in vitro [60], and the nucleotides upstream of the cleavage site are less structured than surrounding sequences [59]. Lastly, RNA-seq based analysis of SOX cleavage sites suggests that the nuclease does not have a consensus target sequence, and rather uses a degenerate sequence pattern to guide mRNA cleavage [59]. Indeed, the lack of a specific targeting motif likely allows SOX to cleave a larger number of host mRNAs.

Microarray analysis of host mRNAs after lytic infection of KSHV revealed that several mRNAs, including IL6, escape decay [61]. Further work demonstrated that up to one-third of cellular mRNAs can escape SOX-mediated degradation [58, 62]. IL6 has a dominant escape mechanism conferred by a 200 nucleotide sequence that recruits RNA-binding proteins to shield the IL6 transcript from SOX [63, 64]. The sequence, called the SOX resistance element (SRE), blocks RNA degradation by the other γ-herpesvirus nucleases, Vhs, a non-homologous α-herpesvirus nuclease, as well as an influenza A virus endonuclease (PA-X) [64]. Addition of the SRE to a GFP reporter inhibits degradation of GFP mRNA, indicating that the SRE is sufficient to confer escape [64]. During infection, IL-6 is also N6-methyladenosine modified in the SRE, which is essential for its evasion of SOX [65]. Interestingly, the 3ʹ UTR of C19ORF66, or Shiftless, can confer resistance to the SOX nuclease, as well as the viral nucleases vhs, mSOX, and BGLF5 [66]. This is particularly interesting since the Shiftless protein plays an antiviral role by limiting programmed frameshifting. The molecular mechanism by which the SRE, or the 3ʹ UTR of the C19ORF66 mRNA, protect mRNAs from these nucleases is not yet determined.

## Orthomyxoviruses

**Fig. 2:**
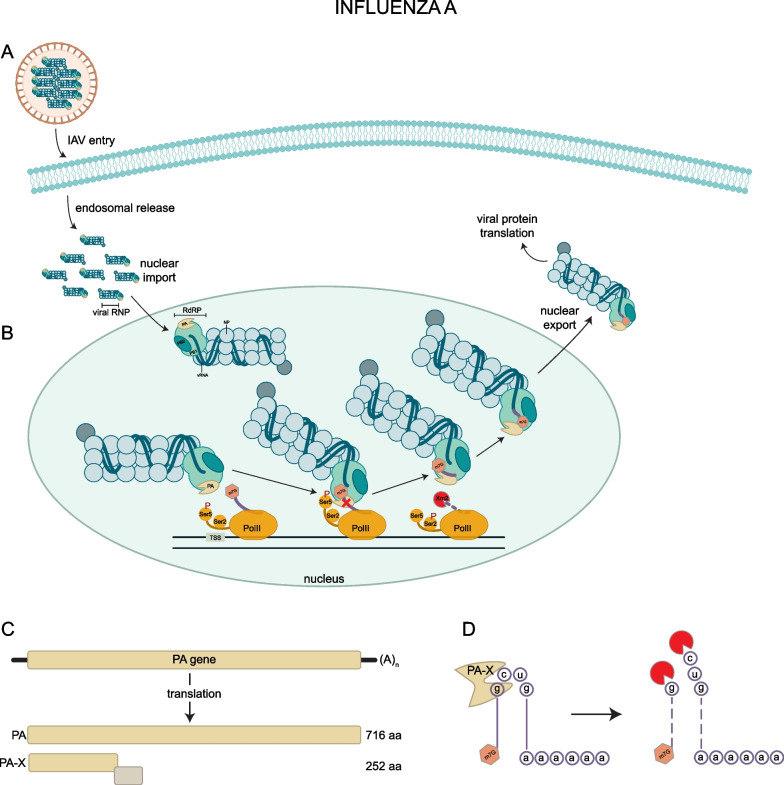
IAV Cap-Snatching and PA-X. (1) IAV particles package eight vRNPs that each include a segment of the viral RNA bound to nuceloproteins (NPs) and the viral RdRp. These vRNPs are imported into the nucleus **B** where they interact with RNA PolII to snatch the caps off of nascent host mRNA transcripts. **C** The endonuclease PA-X arises from a ribosomal frameshift in the PA gene. **D** PA-X preferentially cleaves the GCUG motif and the resulting mRNA fragments are further digested by exonucleases

The orthomyxoviruses include the influenza A viruses (IAV), which are negative sense single-stranded RNA viruses that infect roughly 10% of the adult population and nearly 20% of the pediatric population in the United States annually [67]. One well-established mechanism of IAV host shutoff is via NS1, which has been extensively reviewed [3]. IAV virions consist of 8 segmented single-stranded (negative) RNAs (genomic RNAs), which are each packaged into viral RNPs (vRNPs) with their own RNA-dependent RNA polymerase (RdRP) and nucleoprotein (Fig. 2A) [4]. The RdRP transcribes the viral ssRNA(-) genomic RNAs into mRNAs and replicates the viral genome [68]. The RdRP is able to transcribe a 3ʹ poly(A) tail on viral RNAs, but unlike some other (-)ssRNA viruses, IAV is not capable of capping its own mRNA since it does not encode capping enzymes. Instead, it “cap-snatches” the m7G cap on nascent mRNAs to prime viral transcription [4] and to ensure that viral mRNAs are structurally similar to host mRNAs. After a host mRNA loses its cap, the nascent transcript is degraded in the nucleus by host exonuclease XRN2 [69].

Mechanistic analyses of cap-snatching indicate that influenza RdRP interacts directly with the C-terminal domain (CTD) of the cellular RNA polymerase II (PolII) during early stages of transcription, marked by phosphorylation of Ser5 on the CTD (Fig. 2B) [70]. Chromatin immunoprecipitation (ChIP) experiments show that RdRP binds regions proximal to host gene promoters [71], and this interaction (along with binding viral RNA) causes a conformational change in the RdRP that is amenable to snatching caps [70]. The nascent host mRNA interacts with the RdRP PB2 cap-binding domain, positioning the mRNA in the PA endonuclease site [72]. This endonuclease domain in the RdRP cleaves the mRNA, then the cap-binding domain rotates ~ 90° so that the cleaved cap-fragment is positioned towards the RdRP active site and the viral mRNA [73]. Influenza indiscriminately cap-snatches from RNAP II-transcribed transcripts, and the frequency of cap snatching depends only on RNA abundance [74].

In addition to cap-snatching, IAV can also directly target host mRNAs for degradation by a separate mechanism. This occurs since the PA subunit of the RdRP encodes a + 1 ribosome frameshifting site that gives rise to a shorter polypeptide that retains the N-terminal endonuclease site along with an C-terminal domain, X-ORF (PA-X) (Fig. 2C) [23].

There are several lines of evidence arguing that PA-X can mediate host shutoff through RNase activity. First, expression of PA-X in cells decreases expression of reporter genes, as measured by their protein products, and reduces both actin and IFNβ mRNA levels [23, 75]. Infection with mutant PA-X virus increases morbidity and mortality of IAV infection, with animal models losing weight faster, having a higher load of inflammatory markers, and a heightened humoral response compared to wild-type IAV infection [23, 75]. The effect of mutant PA-X on viral replication varied by strain: the 1918 H1N1 virus was unaffected, while a seasonal H1N1 variant was attenuated by non-functioning PA-X [23, 75]. In some strains, such as in H5N1 and the 2009 H1N1, deletion of PA-X increases viral replication. [76, 77]. This implies that PA-X is significantly involved in suppressing the host innate and acquired immune responses to viral infection, but perhaps it is not essential for viral replication in some contexts. Second, mutating the frameshifting site (FS), the PA endonuclease domain, or introducing a premature termination codon (PTC) all restore gene expression and mRNA levels [23, 75]. Third, PA-X specifically targets both coding and ncRNAs transcribed by RNA Pol II, sparing viral RNAs transcribed by the viral RdRP [78]. Finally, the + 1 frameshifting motif that gives rise to PA-X is highly conserved in most influennza strains, suggesting it plays a crucial role in viral pathogenesis and replication [23]. Notably, there is also increased codon conservation across the PA gene, and synonymous single nucleotide mutations reduced packaging into viral particles [79, 80]. This double hit of promoting viral transcription through cap-snatching while destabilizing host mRNAs contributes to a reduction in host antiviral gene expression that protects IAV [75]. Additionally, the degradation of host mRNAs means that at 8 h post-infection, more than 50% of the mRNAs in the cell are viral mRNAs which account for half of the translation products [81].

Recent data suggest that PA-X cleaves host mRNAs in structure and sequence specific manner (Fig. 2D) [82] and has a preference for spliced transcripts [83]. First, a GCUG motif was identified through 5ʹ RACE as the PA-X preferred cut site, and it is necessary but not sufficient for mRNA cleavage [82]. PA-X also preferentially cuts single-stranded RNAs, either in hairpin loops or in unpaired stretched [82]. This target specificity potentially highlights a new mechanism by which IAV protects its own mRNAs from degradation, as there are very few GCUG stretches in IAV genomes [82]. Additionally, PA-X preferentially cleaves transcripts with introns, and PA-X cleavage is inversely correlated with exon number [83]. The proposed mechanism is that PA-X interacts with splicing machinery in the nucleus to target nascent transcripts for degradation, and that this also provides another way for IAV to distinguish host mRNAs from viral mRNAs because most IAV mRNAs are not spliced [83].

## Bunyaviridae

Bunyaviruses, similar to orthomyxoviruses, are large (-)ssRNA viruses with a segmented genome. Similar to IAV, the viral genome segments are packaged into vRNPs, which each include the nucleoprotein and the ‘Large’ (L) protein. The L protein contains an RdRP [84]. Each of the bunyavirus genera have been shown to employ a cap snatching mechanism similar to IAV, resulting in host RNA degradation [85, 86]. Many of the individual bunyaviruses encode an PD-(D/E)XK endonuclease motif in the N-terminal domain of the L protein, but as of this writing there is no evidence for a ribosomal frameshifting event that would isolate the endonuclease from the rest of the L protein. Similar to IAV and herpesviruses, bunyavirus infection also leads to PABP translocation to the nucleus [87], which occurs when there is widespread cytosolic host mRNA degradation [88].

## Vaccinia virus (& other dsDNA viruses)

**Fig. 3:**
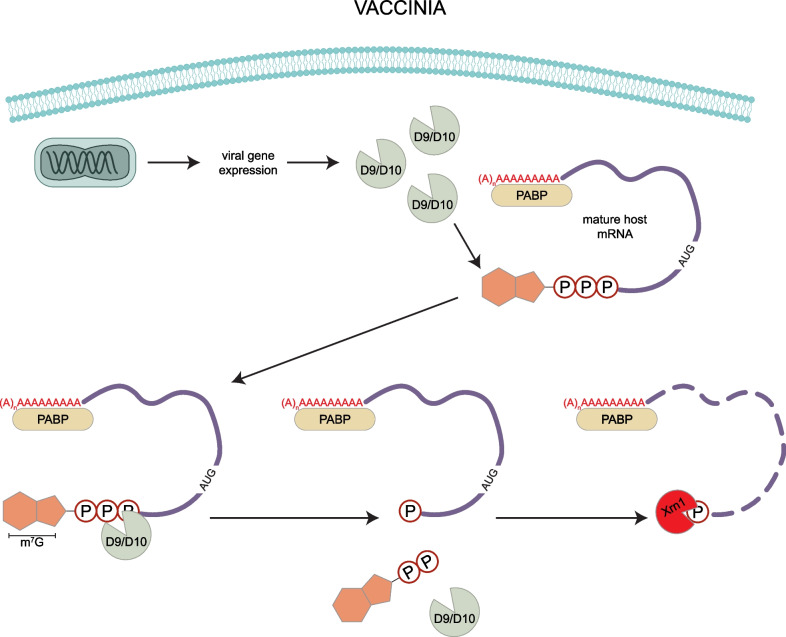
VacV decapping. VacV expresses the proteins D9 and D10 which hydrolyze the m^7^GTP cap on mRNAs, similar to the host decapping enzyme Dcp2. After decapping, mRNAs are digested by the exonuclease Xrn1

Poxviruses are a family of large dsDNA viruses that exclusively replicate in the host’s cytoplasm (Fig. 3). Two examples of poxviruses are Variola virus (VarV), the pathogen that causes smallpox, and Vaccinia virus (VacV) [89]. VacV is closely related to VarV and is therefore used as a laboratory model of the pathogen.

VacV infection was observed to cause degradation of 50% of actin and α-tubulin mRNAs 3 h post-infection [90], and a screen of VacV ORFs identified the proteins D9 and D10 as inhibitors of gene expression at both the protein and mRNA level [91]. D9 and D10 share about ~ 25% of their amino acid sequences, though D10 has a stronger effect on host mRNA levels [91]. Both of these proteins are well conserved across poxviruses, with D10 homologs present in all poxviruses [92, 93]. D10 colocalizes with mitochondria via a three amino acid motif on its N terminus, which is required for both its localization and ability to remove 5ʹ caps [94]. Overexpression of D9/D10 during viral infection inhibited viral protein synthesis [91], suggesting that viral mRNAs are not able to readily escape virally mediated mRNA degradation. However, EMCV IRES reporter mRNAs escape translation repression based on D9/D10 expression (as measured by β-galactosidase activity). This implies that only capped mRNAs or cap-dependent translation are targeted by D9/D10 [91].

Consistent with the observation that VacV host shutoff is related to capped mRNAs, D9 and D10 are sufficient to hydrolyze the m7G cap of a reporter mRNA in vitro (Fig. 3) [95]. Both contain a Nudix/MutT motif, which hydrolyzes nucleoside triphosphates [96]. Notably, the decapping protein Dcp2 also contains a Nudix/MutT motif which is necessary and sufficient for its ability to decap mRNAs [97]. The Nudix/MutT motif forms a loop-α helix-loop structure that binds Mg^2+^ which is required for hydrolysis [98, 99]. Mutating conserved glutamate residues in the Nudix/MutT motif in Dcp2 and in the VacV proteins D9/D10 is sufficient to abolish the hydrolase activity of these enzymes and inhibit decapping [95, 100]. Similar to Dcp2, D9 and D10 both destabilize mRNAs by decapping, which exposes their 5ʹ ends for degradation by the exonuclease Xrn1 as in uninfected cells [101]. VacV mutants lacking D10 degrade host mRNAs more slowly, as measured by Northern blot at different time points post infection [102]. The D10 deletion mutant virus also replicates more slowly than wildtype even though the viral mRNAs are stabilized [102], implying that D10 coordinates viral mRNA expression in addition to host shutoff. Interestingly, when D10 is expressed in uninfected cells, it stimluates translation of mRNA with a 5ʹ-poly(A) leader sequence, indicating a mechanism for maintaining viral translation during host shutoff [103]. D9 and D10 have been shown to target similar viral transcripts, but D10 is primarily responsible for the depletion of human mRNAs, notably targeting transcripts that have undergone splicing [104].

Beyond their role in destabilizing host mRNAs by decapping, D9/D10 are also involved in limiting the accumulation of viral double-stranded RNA (dsRNA), which is recognized by the cellular innate immune response and leads to activation of PKR and RNase L [105]. Infection with VacV containing catalytically dead D9 and D10 lead to reduced expression of late VacV genes, cleaved rRNA, and phosphorylated PKR and eIF2α, consistent with the cellular dsRNA response [105]. Consistent with this observation, Xrn1 depletion during VacV infection slows viral replication, since Xrn1 limits dsRNA accumulation and so blocks PKR activation [106]. This implies that VacV is dependent on Xrn1 to both process decapped host mRNAs and to inhibit the dsRNA sensors that activate promiscuous RNA cleavage by RNase L [106]. Additionally, the high resolution crystal structure of D9 revealed that D9 recognizes caps in a manner distinct from Dcp2, which allows D9 to also recognize and bind the 5ʹ caps of dsRNA [98].

In addition to targeting host mRNAs, VacV also destabilizes host miRNAs through non-templated polyadenylation and degradation [107]. VacV infection led to a 30-fold reduction in all endogenous miRNAs without affecting other small RNAs [107]. Sequencing analysis of small RNAs (20-35nt) after VacV infection revealed that all host miRNAs were poly(A) tailed with 7–9 additional adenosines on the 3ʹ end, and these tails induce degradation of the miRNA. VacV encodes its own poly(A) polymerase to tail viral mRNAs, and the catalytic subunit of this polymerase, VP55, is both necessary and sufficient for oligo(A)-tailing host miRNAs. 3ʹ terminal methylation of miRNAs is sufficient to inhibit VP55 from adding oligo(A) tails to miRNAs and therefore protects against degradation. Altering the miRNA landscape in infected cells would have the downstream effect of massively altering the host transcriptome.

## Coronaviruses

Coronaviruses are some of the largest RNA viruses, with a ( +) ssRNA genome up to almost 33 kb. Of the four genera of coronaviruses, only α-CoV and β-CoVs are known to infect humans. These are responsible for variants of the common cold (HCoV-229E, HCoV-NL63, HCoV-OC43, and HCoV-HKU1) as well as epidemic and pandemic level respiratory infections (MERS-CoV, SARS-CoV, SARS-CoV-2). Both α- and β-CoVs encode the host shutoff factor Nsp1 [108]. The α-CoV Nsp1 is less well characterized and is different in sequence and structure than that of the β-CoV Nsp1, but the translation inhibition function is conserved [108]. The discussion below will focus on SARS1 and SARS2 Nsp1, since those are the most well-studied.

### What is Nsp1?

**Fig. 4:**
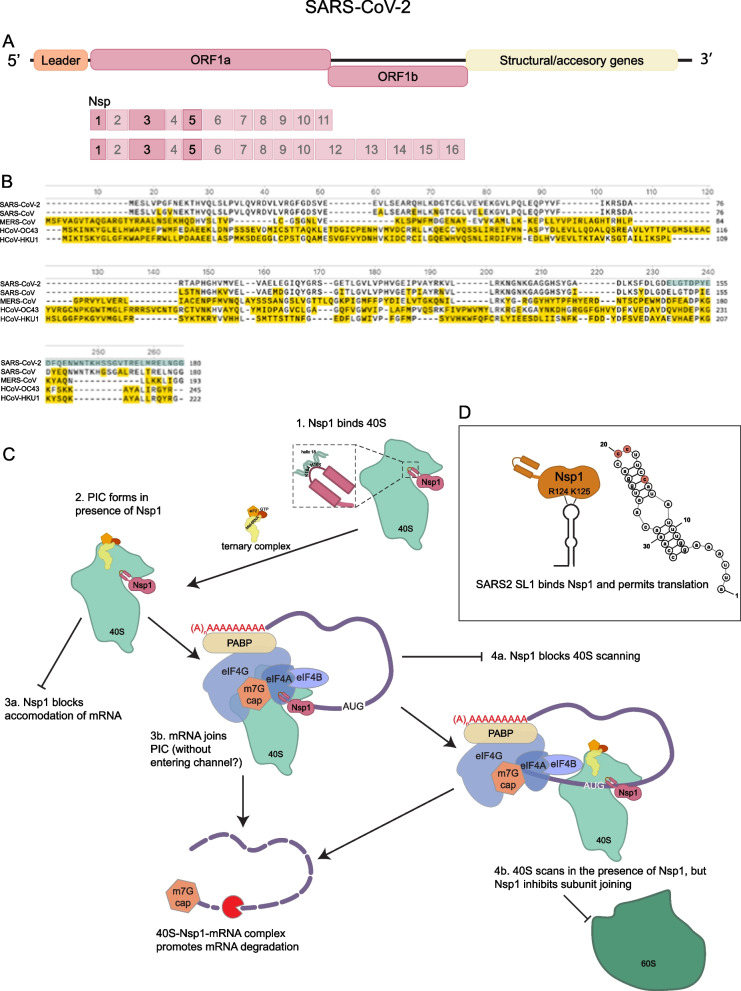
SARS-CoV-2 Nsp1 Host Shutoff. **A** Schematic of the SARS2 genome and location of Nsp1 gene. **B** Conservation of Nsp1 amino acid sequence across different βCoVs. Yellow highlight indicates differences compared to SARS2; green highlight is the CTD. **C** Nsp1 CTD binds the 40S ribosome. Illustrations depict potential Nsp1-40S-mRNA configurations that lead to translation repression and mRNA decay. **D** SARS2 mRNA encodes a stemloop in its 5ʹ UTR that binds to specific resides on the NTD of Nsp1 and allow the viral mRNA to be translated and escape decay

SARS-CoV-2 (SARS2) Nsp1 is encoded by ORF1a/b, which comprises about two-thirds of the SARS2 RNA genome (Fig. 4A) [9]. ORF1a/b is made up of all the non-structural proteins (Nsp), including the RNA-dependent RNA polymerase. The ORF1a/b is translated by cap-dependent translation to produce two polyprotein fusions of ~ 470kD and ~ 760kD [9]. The polyproteins are processed into individual proteins by two viral proteases, Nsp3 and Nsp5.

Nsp1 is the most N-terminal protein to be translated from ORF1a/b, and it is ~ 20kD and contains ~ 180 amino acids. SARS2 Nsp1 is approximately 85% conserved with SARS-CoV-1 Nsp1, though it shares only ~ 26% sequence similarity with MERS-CoV Nsp1 (Fig. 4B) [14, 25, 26]. In general, β-CoV Nsp1s across different lineages share similar functions, despite differences in length and amino acid sequence [108]. Since SARS1 and SARS2 Nsp1 are so similar, much of the foundational research into SARS1 can be applied to SARS2. Both SARS1 and SARS2 Nsp1 are comprised of three different domains: a globular N terminal domain and a helical C terminal domain connected by a short disordered linker [25, 26, 109]. Nsp1 proteins from MERS, SARS1, and SARS2 suppress host translation and also trigger host mRNA degradation [25, 26, 108, 110].

### Nsp1 translation repression

There is abundant evidence asserting that Nsp1 can repress translation (Fig. 4C). First, when recombinant SARS1 or SARS2 Nsp1 is added to in vitro translation extracts, translation is reduced [25, 26, 111]. Importantly, the mRNAs in these extracts are not degraded by Nsp1 demonstrating translation is being directly reduced [112]. Second, when SARS1 or SARS2 Nsp1 is transiently expressed in tissue culture cells, protein production from reporter genes is reduced [26, 110, 112, 113]. Interpreting the reduction in cells is more difficult since transiently expressed Nsp1 can also trigger mRNA degradation of host [110, 113], and transfected reporter mRNAs [25, 26, 111, 114–116]. Importantly, variants of SARS2 Nsp1 have been identified that limit degradation of host or reporter mRNAs but still show translation repression [113, 114]. This provides additional evidence that Nsp1 can repress translation independently of its ability to degrade cellular mRNAs.

Several observations argue translation shutoff by Nsp1 requires interaction with the small ribosomal subunit (Fig. 4C). Eukaryotic translation initiation requires the formation of the pre-initiation complex, with 40S (small ribosomal subunit) and initiation factors recognizing an mRNA. The 40S subunit will then scan an mRNA until recognition of an initiation codon, whereupon it recruits the 60S subunit and begins translation [117]. The key observations are as follows. First, sucrose gradient fractionation of in vitro translation systems supplemented with recombinant Nsp1 and cells expressing Nsp1 both show that Nsp1 co-migrates with the 40S subunit [25, 26, 112]. Moreover, when expressed in cells, Nsp1 immunoprecipitates with several 40S ribosomal proteins, such as RACK1, RPS2, and RPS3 [114]. Additionally, Nsp1’s interaction with the ribosome is abolished when two C-terminal residues (K164 and H165) are mutated [25, 26, 111, 112, 114]. Abolishing the Nsp1-40S interaction alleviates translation repression both in cells and in vitro [25, 26, 113, 114], demonstrating that Nsp1 blocks host translation through a direct interaction with the small ribosomal subunit.

Two cryo-EM structures of Nsp1 provide insights into how SARS2 Nsp1 interacts with the 40S ribosome and mediates host shutoff [25, 26]. Both observed that the C-terminal α-helices of Nsp1 bind tightly to the mRNA entry channel, completely obscuring the mRNA path. These α-helices interact with the rRNA helix h18 and ribosomal proteins uS3 (head) and uS5 (body) and mutating the residues that directly interact with uS3 and uS5 blocks translation repression [25, 26]. The KH motif is part of a short loop connecting the two α-helices, and it interacts with the 530-loop of helix h18, part of the ribosome decoding center [25, 26, 118]. In addition to describing the Nsp1-40S interactions, these structural studies also found 43S pre-initiation complexes (PICs), some including the eIF2-tRNA_i_-GTP ternary complex, bound to Nsp1, demonstrating that Nsp1 permits complete 43S formation. The way that Nsp1 interacts with the 40S has led to the model that translation is repressed by sterically obstructing mRNA loading, changing the conformation of the 40S, and potentially interfering with ribosome decoding [25, 26, 119].

Surprisingly, the structural analyses also revealed Nsp1 in complex with 80S ribosomes. This poses a conundrum, because SARS1 Nsp1 was shown to inhibit subunit joining in vitro [112], and 80S-cricket paralysis virus (CrPV) internal ribosome entry site (IRES) complexes inhibit rapid Nsp1 binding to the 40S subunit [119]. Most of the 80S-Nsp1 structures were unusual and previously undescribed, and none of them contained mRNA [26]. One sub-group of 80S-Nsp1 complexes included the protein CCDC124, which is indicative of an idle state prior to ribosome recycling [120]. Supporting the hypothesis that 80S-Nsp1 is an intermediate prior to ribosome disassembly, one subpopulation included both CCDC124 and ABCE1, a canonical ribosome quality control factor that splits 80S ribosomes into the 60S and 40S subunits [121].

The prevailing interpretation of the above observations is that when Nsp1 is bound to the 40S ribosome, mRNA cannot be accommodated properly so that a 40S-mRNA complex is impermissible. This is supported by two experimental observations. First, the CrPV IRES binds predominantly to the mRNA exit site in the presence of 40S-Nsp1 [115]. The cryo-EM structure of the IRES loaded on to the 40S in the presence of Nsp1 shows that the mRNA could not displace Nsp1 from the mRNA entry channel. While the CrPV IRES is a useful tool for studying a simplified model of translation initiation without eIFs [122], eIFs have been shown to allosterically regulate the Nsp1-40S assembly in the absence of mRNA [119], so conclusions from the CrPV-IRES-40S-Nsp1 structure are limited. Second, single molecule fluorescence assays demonstrate that mRNA directly competes with Nsp1 for binding at the mRNA entry channel and that the presence of either Nsp1 or mRNA on the 40S precludes the other from binding efficiently [119]. Specifically, the observation is that there is a dose-dependent response between the length of the mRNA downstream of the start codon and the percent of Nsp1 that can stably bind the 40S [119] because more of the mRNA entry channel is filled with mRNA. However, even the least efficient conditions for Nsp1 binding—eIFs and an mRNA with 41 nucleotides downstream of the start codon—still permitted 16% of present Nsp1 to bind the 40S [119]. These experiments were carried out in vitro with 25 nM of Nsp1, but in the context of a viral infection Nsp1 is expected to be at much higher concentrations.

Nsp1 is a potent translational repressor that interacts with the open state of the 40S ribosome, likely impeding normal initiation either by blocking mRNA accommodation, preventing subunit joining, or by promoting ribosome turnover [25, 26, 115, 119]. Previous polysome profiling experiments indicated that SARS1 Nsp1 does not inhibit 48S formation in RRLs, but it does block 80S formation [112]. However, it is unclear exactly what Nsp1-40S-mRNA assembly forms in cells or if the 48S complex observed in RRL resembles a functional 48S.This is important as this complex likely plays a role in mediating mRNA decay (discussed below).

## Viral mRNA escape of Nsp1 translation repression

Host shutoff via Nsp1 presents a paradox for viral replication: when all of the host ribosomes are blocked from forming proper initiation complexes, how does the viral mRNA get translated, particularly if the 40S subunit is trapped in a conformation not amenable to mRNA loading?

Several observations suggest that the escape mechanism for the viral mRNA involves the structure and sequence of the 5ʹ leader that is present on all viral genomic and subgenomic RNAs (Fig. 4D) [123]. The leader sequence on SARSs mRNAs is 70nt and is predicted to contain three stem loops [124]. The first stem loop (SL1) has been shown to be necessary and sufficient for mRNA escape from translation repression [114, 125, 126]. SL1 contains two 10 bp double stranded helices separated by a bulge in the middle, with a 4nt loop at the top. When SL1 precedes a reporter mRNA sequence, that mRNA is both protected from degradation [114] and is translated normally [125–127]. Interestingly, one study observed that mRNAs with the leader sequence are preferentially translated in the presence of Nsp1, generating up to 3 × more protein than when Nsp1 is not present [125]. When SL1 is mutated or removed from the leader sequence of a reporter mRNA, translation repression is restored [125]. Additionally, ASOs targeting SL1 restore SARS2 mRNA susceptibility to translation repression [125, 126]. Taken together, these observations demonstrate that SL1 is the critical feature of the viral leader sequence that mediates viral escape from host shutoff. NSP1 was recently shown to cleave host and viral transcripts as they are loaded onto the ribosome, with unique cleavage patterns dependent on the target RNA [128]. This suggests that NSP1 is an endonuclease that works in conjunction with the ribosome to cleave target mRNAs [128]. This discovery of differential cleavage sites may help explain how SARS2 can promote host shutoff while preserving viral transcripts.

SL1 confers escape from Nsp1 through its sequence, not just its structure (Fig. 4D). Two cytosine residues on the loop (C19 and C20) and one cytosine on the stem proximal to the loop (C15) are all necessary for translation to proceed [125]. Mutating the stem sequence but maintaining the structure leads to translation repression in the presence of Nsp1 [125, 127]. This suggests that SL1 interacts in a sequence-specific manner with Nsp1. Three N-terminal residues on Nsp1 are required for interacting with the SARS2 leader sequence [114]. However, the nature of the Nsp1-SL1 interaction is still unknown, and how the presence of SL1 changes the Nsp1-40S blockade is unclear.

Interestingly, other viral mRNA structures can also confer resistance to Nsp1. Two flavivirus IRESes (from HCV and CSFV) were not endonucleolytically cleaved by in RRL supplemented with SARS1 Nsp1 [112]. This is in contrast to IRESes from picornaviruses, which show distinct cleavage products in both RRL and when expressed in cells and have premature termination products in primer extension analyses [129].

### Nsp1-mediated mRNA decay

In addition to repressing host cellular translation, there are many observations that Nsp1 can also promote the degradation of endogenously expressed host and transfected reporter mRNAs (Fig. 4C). First, transient expression of SARS1 Nsp1 was observed to reduce IFN-β and reporter mRNA levels in cells, independently of treatment with actinomycin D, a transcription inhibitor [24]. During viral infection, host cell mRNA degradation occurs rapidly, within 8 h, and is independent of RNase L [110]. Interestingly, Nsp1 does not cause mRNA degradation after IRES cleavage in RRL, although primer extension and toe-printing analyses show impaired start codon recognition and possible removal of the 5ʹ cap [112], which could be consistent with Nsp1 triggering cleavage of ribosome bound mRNAs [128], but RRL lacking the nuclease that degrades mRNAs. All of the residues necessary for only stimulating mRNA degradation are in the N terminal domain of Nsp1 [114, 130], suggesting that perhaps the NTD stabilizes an interaction between the mRNA and a host ribonuclease.

Translation repression and mRNA decay are two separate functions of Nsp1, as demonstrated by separation of function mutations in which translation is off but mRNA is not degraded [113, 114, 130]. However, mRNA degradation requires an Nsp1-40S assembly: all of the C terminal mutations that disrupt Nsp1 binding to the ribosome also disrupt mRNA degradation [112, 114, 130]. This illustrates that mRNA decay is downstream of translation repression but is a separable molecular event.

Supporting this idea is the observation that only translated RNAs are degraded by Nsp1. Long non-coding RNAs (lncRNAs) are unaffected by Nsp1 expression[113]. Moreover, there is a strong correlation between mRNA translation efficiency and Nsp1-mediated RNA degradation, suggesting that an mRNA must interact the Nsp1-40S assembly [131], consistent with Nsp1 cleaving mRNAs in association with the ribosome [128]. However, the exact nature of how an mRNA would interact with an Nsp1-40S complex remains to be determined.

Another open question is the mechanism of mRNA decay. Nsp1 alone does not have any nuclease activity and bears no resemblance to known ribonuclease domains [129, 132]. Furthermore, Nsp1 cannot bind mRNA directly [114, 127]. SARS1 Nsp1 can induce endonucleolytic cleavage in some IRES structures in RRLs [112, 129, 130]. Specifically, Nsp1 cleaves the EMCV IRES and several picornavirus type I and II IRESes, but not the CrPV or flavivirus IRESes, such as hepatitis C IRES or CSFV IRES [112, 129]. These cleavage events occur in the 5ʹ UTR of the mRNA, about 30nt downstream of the cap. Since the ribosome footprint is 30nt, this suggests that the mRNA is cleaved near a potentially bound ribosome [129]. This implies that perhaps ribosomes could stall and collide when Nsp1 is present, possibly recruiting ribosome quality control pathways and their associated endonucleases.

After an mRNA is cleaved by an endonuclease, 5ʹ-3ʹ and 3ʹ-5ʹ exonucleases will degrade the fragments. Therefore, if Nsp1 causes endonucleolytic cleavage of mRNAs, these cleavage products are likely processed by exonucleases as well. This was tested by knockdown of Xrn1, the predominant 5ʹ-3ʹ exonuclease in mammalian cells. Loss of Xrn1 stabilizes reporter mRNAs that were degraded by Nsp1 by about 20% [39], suggesting Xrn1 may be involved in processing Nsp1 degradation products. However, the modest stabilization phenotype implies that there are probably other unidentified nucleases involved. Proximity-labeling the Nsp1 interactome identified DIS3, a component of the RNA exosome, and Xrn2 as potential candidates for mediating mRNA decay [125], but they have yet to be tested in cells expressing Nsp1.

## Consequences of host mRNA degradation

**Fig. 5:**
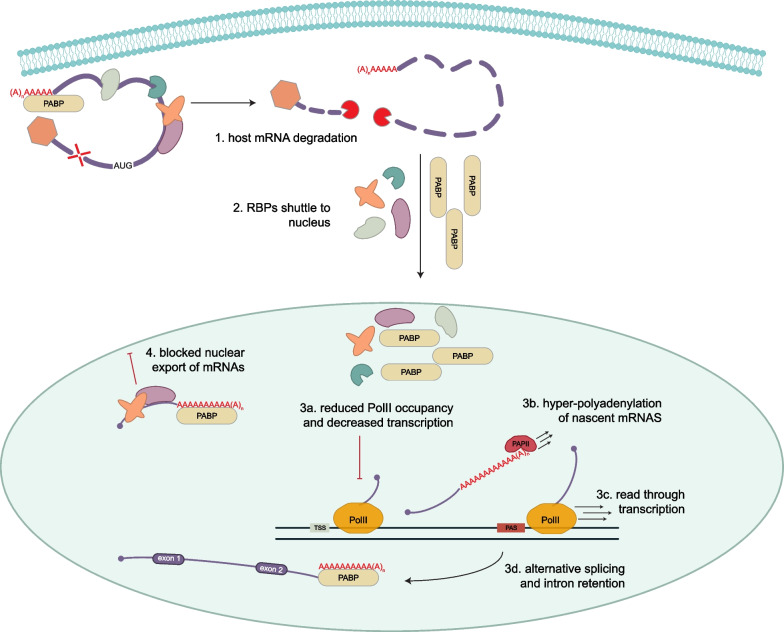
Consequences of Cytosolic mRNA Decay. Widespread cytosolic mRNA decay causes RPBs to shuttle to the nucleus, where they reduce RNA PolII occupancy and transcription; cause hyper-adenylation of mRNAs (which contributes to nuclear retention); lead to read-through transcription and alternative splicing. All of this leads to an export block of mRNAs, so that mRNAs are trapped in the nucleus

In addition to the degradation of host mRNAs, there are additional consequences to the cell when cytosolic mRNAs are substantially degraded. These consequences occur in response to diverse types of cytoplasmic mRNA degradation during viral infection and include increased partitioning of cytosolic RNA binding proteins (RBPs) in the nucleus, alterations in RNA processing, altered mRNA export, reduced translation, and reduced transcription (Fig. 5).

### Cytoplasmic RNA degradation re-localizes RNA binding proteins in the nucleus

An important set of observations argued that widespread RNA decay could lead to cytosolic RBPs concentrating in the nucleus. Specifically, it was observed that expression of the herpes virus nucleases SOX, muSOX, or vhs cause PABPC, and other RBPs, to accumulate in the nucleus [133–135]. Similar results are seen when RNase L is activated, which triggers widespread cytosolic mRNA degradation [21, 22], leading to the nuclear concentration of PABPC and other cytosolic RBPs [22, 88]. Similarly, dengue virus infection and SARS2 infection also lead to PABP nuclear influx following RNase L activation [88]. Exogenous expression of Nsp1 can also cause PABP to translocate to the nucleus, though less dramatically than SOX and muSOX [113]. Notably, PABP translocation to the nucleus was only observed in SARS2-infected cells that express and activate RNase L [110], indicating that nuclear PABP translocation during SARS2 is largely host-mediated. This could imply that SARS2 limits PABP translocation to the nucleus and/or that the abundant viral mRNAs bind PABP, thus limiting nuclear PABP translocation. These experiments all suggest that widespread cytosolic RNA degradation leads to increased nuclear concentrations of RBPs.

Several observations suggest that the nuclear concentration of RBPs after cytosolic RNA decay is due to the nuclear-cytoplasmic shuttling of RBPs, and then increased retention in the nucleus when RNA is only present in the nucleus. This possibility is supported by the observation that concomitant degradation of nuclear and cytoplasmic RNA repartitions RBPs back to the cytosol [88]. Consistent with the interpretation, the loss of nuclear RNAs by inhibiting transcription leads to the movement of RBPs to the cytosol [136], and the exposure of additional cytosolic mRNA sequences during a stress response when ribosomes run-off the majority of mRNAs [137].

### RNA binding proteins translocate to the nucleus and alter RNA transcription and processing

Increasing nuclear RBPs has several consequences, including altered transcription, RNA processing (including splicing and poly(A) tail length), and inhibiting RNA export. For example, the translocation of PABPC following SOX-mediated cytoplasmic RNA decay to the nucleus can initiate hyperadenylation of mRNAs, leading to an mRNA export block [134, 135]. The key observations are that SOX expression leads to increased poly(A) tail length and increased nuclear poly(A) mRNA, in a PABPC-dependent manner [134, 135]. Additionally, mass spectrometry of nuclear fractions taken during gammaherpesvirus infection showed an nuclear accumulation of RBPs, which was dependent on the cleavage activity of muSOX [9].

Widespread cytosolic mRNA degradation following RNase L activation also leads to altered RNA processing. In this case, RNA sequencing of WT and RNase L knockout (RLKO) cells treated with mock or poly(I:C) identified 140 differential splicing events across 136 genes [88]. Additional analyses revealed an increase in retained introns in many genes that passed filtering after poly(I:C) treatment, which was validated by smFISH. It is still unclear why some genes, like GAPDH and ACTB, do not retain introns after RNase L activation while other genes do, though this may be a result of differential transcription rates during the dsRNA response.

Host shutoff also disrupts transcription termination. This has been observed during lytic HSV-1 infection, IAV infection, and SARS2 infection. For example, 4SU-labeling of nascent RNAs and subsequent RNAseq 8 h post-HSV1 infection revealed substantial reads downstream of transcription stop sites [16]. Moreover, IAV disrupts poly(A) signal-dependent termination separately from its host shutoff function through the viral NS1 protein [138]. This is hypothesized to occur by NS1 interacting with and inhibiting the function of the 3ʹ termination processing protein CPSF, which in turn leads to read-through transcription as observed by RNAseq [138–140]. Read through transcripts are primarily retained in the nucleus, which could explain the impact on mRNA export [141].

These downstream of gene transcripts (DoGs) have also been identified in response to osmotic stress and RNase L activation after viral infection or poly(I:C) treatment [88, 142]. Interestingly, IFNβ1 DoGs induced by RNase L activation are diffuse throughout the nucleus [88] and do not remain localized to the chromatin transcription site [143]. They are also not exported to the cytoplasm and appear to inhibit normal IFNβ1 from exiting the nucleus through an unknown mechanism. These effects on RNA processing appear to be independent of PABPC accumulation the nucleus [88], which is different from the defect in mRNA export triggered by SOX mediated RNA decay [134]. Analysis of nuclear:cytoplasmic PABPC intensity does not correlate with nuclear IFNβ1 mRNA retention and trapping PABPC in the nucleus with a nuclear retention signal does not lead to retained IFNβ1 mRNAs [88]. Additionally, Xrn1 knockdown prior to RNase L activation retains PABPC in the cytoplasm, while cells still exhibit DoG production and a defect in export of the IFNβ1 mRNAs [88]. Together, these data raise the possibility that translocation of PABPC to the nucleus may not be sufficient or necessary for trapping mRNAs in the nucleus under all conditions of cytoplasmic RNA degradation.

Importantly, cytoplasmic mRNA degradation during viral infection can suppress cellular RNAPII transcription [144–146]. Expression of host shutoff proteins that trigger host RNA decay reduces RNAPII occupancy by ChIP and transcription as measured by 4SU labeling, but the RNA decay function must be intact for this to occur [144]. Additionally, RNA decay enzymes are required for reduced transcription: knockdown of the RNA exosome, exonucleases, or deadenylation enzymes in the context of host shutoff restores RNAPII occupancy on chromatin [133, 144]. Notably, this is opposite to how cells normally respond to cases of reduced RNA stability, when they increase transcription to maintain normal gene expression [145]. However, during apoptosis mRNA decay represses RNAPII [147]. Expression of host shutoff proteins drives a different cellular response, perhaps because of the rapid global degradation of cellular mRNAs.

Transcriptional suppression is likely also driven by RBPs shifting to the nucleus. Nuclear PABPC1 is sufficient to block PolII recruitment to promoters [133]. One possible mechanism is that when cytoplasmic RBPs are in the nucleus at sufficient local concentrations, they can disrupt the formation of the transcriptional pre-initiation complex by disrupting other RBP or chromatin-binding protein partners [133]. Additional evidence for RBPs affecting transcription comes from the observations that many RBPs associate with chromatin and affect transcriptional output [148].

### Cytoplasmic decay can alter nuclear export of mRNAs

The widespread decay of cytosolic mRNAs can also lead to alterations in mRNA export. This was first suggested by the observations that degradation of host mRNAs by SOX led to decreased mRNA export [134, 135]. Similarly, widespread RNase L degradation leads to a block to mRNA export [110, 149].

One anticipates two possible mechanisms by which cytosolic RNA decay will alter mRNA export. First, the alterations in poly(A) tail length [134, 135], intron retention [88], and 3ʹ end extensions [88] can create mRNAs that are recognized as not fully mature and are thereby retained in the nucleus [150]. Alternatively, the increased concentration of RBPs in the nucleus might compete for export factors binding the mRNA leading to nuclear retention. In some cases, other viral proteins might directly inhibit export. For example, SARS2 Nsp1 is suggested to bind to the mRNA export factor NXF1 and reduces NXF1 association with the adaptor Aly/REF, which required for recruiting the NXF1-NXT1 export heterodimer [116, 151]. Nsp1 also interacts with the upstream helicase UAP56, which recruits Aly/REF to the mRNA. Together, this suggests that Nsp1 might also interfere with mRNA export by inhibiting a functional NXF1-mRNA complex. Supporting this, SARS2 infected cells have less NXF1 on the nuclear envelope than mock infected cells, and overexpressing NXF1 reduced the number of infected cells [116].

## Conclusions and outstanding questions

Many viruses have evolved mechanisms to prevent the host antiviral response and to ensure that viral proteins are preferentially translated. While there are examples of host-shutoff only targeting translation, here we’ve described the mechanisms by which viruses degrade host mRNAs. Diverse families of virus degrade host mRNAs—both sense and antisense ssRNA viruses, as well as dsDNA viruses—yet there are some commonalities. First, many of these viruses encode an endonuclease that cleaves ssRNA, which is then processed further by the host RNA exosome and the 5ʹ–3ʹ exonuclease Xrn1. Second, there is some degree of specificity: viruses (with the exception of HSV) preferentially target motifs or structural elements that are common in the host genome but absent from viral mRNAs, this sparing the viral mRNAs from degradation. Similarly, SARS2 mRNA encodes a structure that resolves the Nsp1-mediated translation inhibition and RNA degradation.

While this field of viral-induced host-shutoff is quickly evolving, there are still several open questions. First, the mechanism by which virally encoded nucleases target specific host mRNAs is still unclear. There is data supporting the hypotheses that these nucleases target RNAs that are unpaired or part of an open loop, and that RNAs are targeted during an early stage of translation. Likewise, it is becoming clear that more host mRNAs are escaping host-shutoff more than previously thought. For example, host antiviral mRNAs (i.e., type I interferon) evade both RNase L- and Nsp1-mediated mRNA decay during SARS2 infection [22, 110]. Thus, the landscape of mRNA destabilization is more likely more nuanced than realized. Moreover, host and viral mRNAs might be undergoing an evolutionary arms race to evolve structures that modulate their stabilization during host- and viral-mediated mRNA decay. Thus, it is early days in our understanding differential mRNA stability during viral infections.

Additionally, massive cytosolic host mRNA degradation has significant impacts on the subcellular localization of RBPs. It appears that there are specific effects on certain mRNAs and RBPs in response to different initiators of mRNA degradation. The mRNA nuclear export block in response to SOX/muSOX overexpression is dependent on PABPC translocation to the nucleus, whereas the export block in response to RNase L activation in the cytosol is independent of PABP. This suggests that there are unexplored effects of different RBPs and different mechanisms by which distinct viruses/antiviral responses lead to the same outcome in the cell. Moreover, understanding the molecular mechanisms and specificity of viral host shut-off mechanisms has the potential to lead to new antiviral strategies and immunomodulatory therapies.

## Data Availability

Data sharing is not applicable to this article as no datasets were generated or analysed during the current study.
